# A broadly distributed species instead of an insular endemic? A new find of the poorly known Hainan gymnure (Mammalia, Lipotyphla)

**DOI:** 10.3897/zookeys.795.28218

**Published:** 2018-11-05

**Authors:** Alexei V. Abramov, Anna A. Bannikova, Vladimir S. Lebedev, Viatcheslav V. Rozhnov

**Affiliations:** 1 Zoological Institute, Russian Academy of Sciences, Universitetskaya nab. 1, Saint Petersburg 199034, Russia; 2 Joint Vietnamese-Russian Tropical Research and Technological Centre, Nguyen Van Huyen, Nghia Do, Cau Giay, Hanoi, Vietnam; 3 Lomonosov Moscow State University, Vorobievy Gory, Moscow 119992, Russia; 4 Zoological Museum of Lomonosov Moscow State University, B. Nikitskaya 6, Moscow 125009, Russia; 5 A.N. Severtsov Institute of Ecology and Evolution, Russian Academy of Sciences, Leninskii pr. 33, Moscow 119071, Russia

**Keywords:** Distribution, *
Neohylomys hainanensis
*, new findings, Vietnam

## Abstract

The Hainan gymnure *Neohylomyshainanensis* (Mammalia, Lipotyphla), endemic to Hainan Island (China), is one of the rarest and least-known species within the family Galericidae. The IUCN Red List inferred it as an endangered species due to ongoing population decline caused by natural habitat loss. A recent biodiversity survey has revealed *N.hainanensis* to be rather common in northern Vietnam. This is the first record of the species outside Hainan Island. New data have allowed us to re-assess the conservation status of this poorly known mammal. The occurrence of *N.hainanensis* in mainland Vietnam also supports the hypothesis that Hainan Island could have been previously connected to Guangxi and northern Vietnam rather than to neighbouring Guangdong.

## Introduction

The family Galericidae (Mammalia, Lipotyphla) comprises six recent genera and 8–10 species of gymnures and moonrats inhabiting tropical and subtropical forests of southern China and SE Asia, including the Philippines and the Sunda Islands (Hutterer 2005, Bannikova et al. 2014). The majority of its species are listed as endemics, having very small distributional ranges restricted to some islands (*Podogymnuratruei*, *P.aureospinula*, *Neohylomyshainanensis*) or small mountainous areas (*Hylomysparvus*, *Otohylomysmegalotis*).

The Hainan gymnure *Neohylomyshainanensis* Shaw & Wong, 1959 is usually regarded as one of the rarest and least-known species of all the Galericidae (Stone 1995, Johnston and Smith 2016). This species remains known from a few museum specimens only (Stone 1995). The recent IUCN Red List recognized the Hainan gymnure as an endangered species, because it is known from the island of Hainan only, and its range is less than 5,000 km² (Johnston and Smith 2016). According to published data (Corbet 1988, Hoffmann and Lunde 2008), this species occurs in tropical rainforests and subtropical evergreen forests. During the last decades, the forests of Hainan Island have been under considerable anthropogenic pressure due to logging and agricultural use. It is agreed that the population of *N.hainanensis* is in decline due to habitat loss (Johnston and Smith 2016).

## Materials and methods

**Figure 1.**
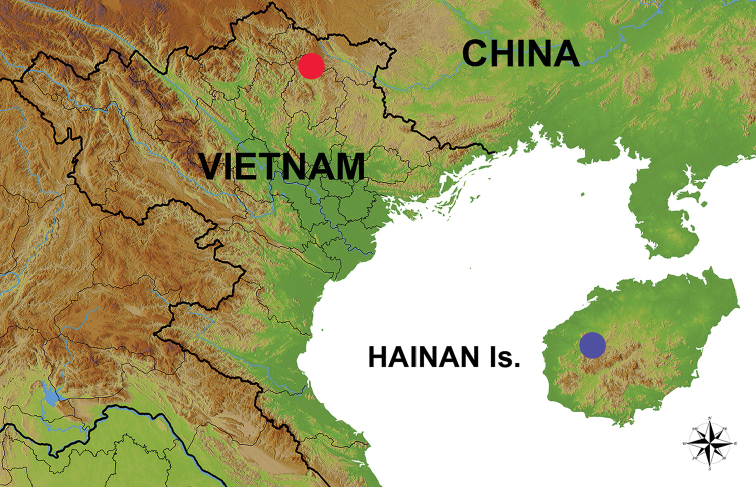
Distribution map of *Neohylomyshainanensis*. Previously known records from Hainan Island are marked with blue, new findings in Vietnam are marked with red.

During the 2018 small mammal surveys conducted by the Joint Vietnamese-Russian Tropical Research and Technological Centre in northern Vietnam, five specimens of small gymnures were collected in Cao Bang Province, approximately 22°37'41"N, 105°54'41"E, at elevation 300–700 m a.s.l. (Figure 1). All specimens were obtained from local villagers during studies of rodent distribution and pest control. Voucher specimens (coll. numbers AVA 18-134, AVA 18-125, AVA 18-136, AVA 18-137, AVA 18-138) are kept in the Zoological Institute of the Russian Academy of Sciences (Saint Petersburg, Russia).

## Results and discussion

**Figure 2.**
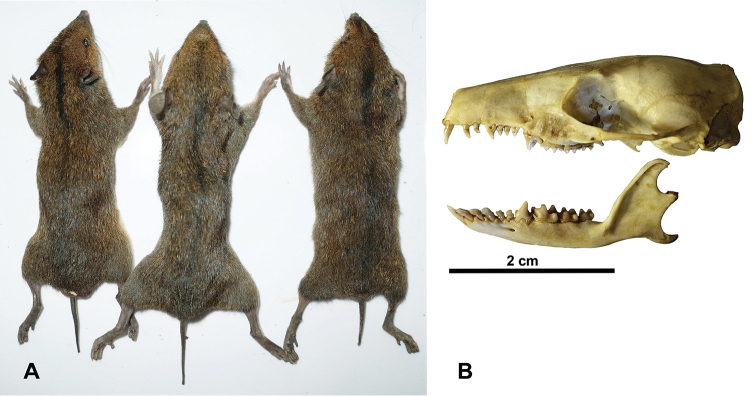
*Neohylomyshainanensis* from Vietnam. **A** total view **B** skull.

A morphological analysis of Vietnamese specimens has revealed their identity as *N.hainanensis*. These are small-sized, vole-like gymnures with a heavily built body and slightly stout, pointed rostrum. Head and body length 120–142 mm, tail length 30–40 mm. Tail is approximately 26.3% of head and body length, whereas it is 70–80% in *Otohylomys*, ca. 50% in *Neotetracus* and 10–15% in *Hylomys*. *Neohylomyshainanensis* from Hainan has its head and body length 120–147 mm, tail length 36–43 mm; relative tail length is 28.9% (Shaw and Wong 1959, Hoffmann and Lunde 2008). Dorsum dull olive-brown; ventral pelage yellowish, lighter than dorsal; there is a longitudinal black line on anterior midback (Figure 2A). Tail bicoloured, dark above and much lighter below. Dental formula: 3.1.4.3/3.1.3.3 = 42. There are four upper and three lower premolars (Figure 2B). First upper incisor is very large. Upper canine teeth only slightly larger than adjacent incisors and premolars. The dentition of Vietnamese specimens in full concordance with that of *N.hainanensis* (Shaw and Wong 1959, Engesser and Jiang 2011).

All the Vietnamese specimens were collected from evergreen mixed forest at the elevations of 300–700 m a.s.l. It was not recorded at higher elevations (1500–1800 m a.s.l. in Phia Oac – Phia Den National Park). According to the information from local villagers, this species is rather common, both in slightly disturbed forests and in primary forests.

In Hainan, the species is recorded from the Jianfengling Nature Reserve and may also occur in the Jiaxi and Wuzhishan nature reserves (Johnston and Smith 2016). The small distributional area and population decline make the species quite vulnerable. In Vietnam, the species has been recorded in and around the Phia Oac – Phia Den National Park in Cao Bang Province (Figure 1). Additional research is needed to estimate the distribution, population and habitat status, and threats to *N.hainanensis* in Vietnam. Little is known about the biology and ecology of this species (Stone 1995). New distribution findings have allowed us to gain additional data on the species’ natural history.

Hainan Island is widely recognized as one of the world’s biodiversity hotspots (Myers et al. 2000). The geological history of Hainan Island, as well as the Indo-Malaysian or East Asian affinity of its biota, is still poorly understood. Some authors suppose it was previously connected to mainland China (Guangdong), whereas others argue that Hainan Island was originally located near Guangxi and northern Vietnam during the early Cenozoic (see Zhu 2016). Vertebrate animal studies have revealed the island to have a higher species diversity and endemism in comparison to adjacent mainland China, which could be related to the geological origin of Hainan (Chen and Bi 2007, Chen 2008, 2009). A comprehensive analysis of seed plant distribution (Zhu 2016) showed that the Hainan flora indeed has a tropical Asian affinity and very low endemism at generic and species levels, which seems to imply its continental origin. Moreover, the Hainan flora shows strong similarities to that of Vietnam and Guangxi, but less so to the adjacent Guangdong Province (Zhu 2016). Our discovery of the Hainan gymnure also supports the idea that Hainan Island could have been connected to northern Vietnam.
